# Supplementary material for: Telmisartan Potentiates Insulin Secretion via Ion Channels, Independent of the AT1 Receptor and PPARγ
Source: Front Pharmacol. 2021 Sep 14;12:739637. doi: 10.3389/fphar.2021.739637 (PMC8477257; doi:10.3389/fphar.2021.739637)
Supplement: Supplementary file 1 [file DataSheet1.PDF]

Supplementary Material

Orally applied valsartan and irbesartan blocked the mean arterial pressure (MAP) response to intravenously injected Ang II

Considering that valsartan and irbesartan, as angiotensin II type 1 receptor blockers, are effective antihypertensive drugs, we evaluated their effects on cardiovascular responses in rats with acute hypertension induced by Ang II to ascertain the bioactivity of valsartan and irbesartan.

Adult male Sprague Dawley rats were divided into three groups (n=6), and the cardiovascular responses were measured by tail-cuff blood pressure meter (BP-2010 Series Blood Pressure Meter, Softron Biotechnology Co., Ltd, Beijing, China). 2 hour after intragastric treatment with the antagonists (10 mg/kg of body weight valsartan or 15mg/kg of body weight irbesartan) or vehicle (control), Ang II was dissolved in isotonic saline and intravenously injected as a bolus (200 ng/kg of body weight). The initial value of arterial pressure and the peak response to each injection was determined, and increases in mean arterial pressure (MAP) were presented as the means ± SEM.

As shown in Fig. S1, injection of Ang II (200 ng/kg of body weight) elicited an immediate, significant increase in MAP of about 45 to 55 mmHg. Both pretreatment with valsartan and irbesartan significantly reduced the MAP response to Ang II injection. The Ang II-induced MAP response was inhibited almost by 50% and 60% with the application of valsartan and irbesartan."

GW9662 inhibited the protective effect of rosiglitazone on glucolipotoxicity-induced damage in INS-1 cell

PPARγ is a member of the nuclear receptor superfamily, and plays important regulatory roles in lipid metabolism. Numerous evidences have indicated PPARγ agonist, including rosiglitazone and troglitazone, exerted protective function on β-cells against glucolipotoxicity-induced cell death (Kawai et al., 2002; Lupi et al., 2004; Walter and Lübben, 2005; Han et al., 2008; Wu et al., 2013). Therefore, we examined whether GW9662 could block the protective effect of rosiglitazone on INS-1 cell exposed to high-glucose and high-lipid condition.

INS-1 cells were obtained from the National Infrastructure of Cell Line Resource (Beijing, China), and were cultured in RPMI 1640 medium containing 11.1 mM glucose, supplemented with 10% fetal bovine serum, 1% penicillin and streptomycin, 10 mM HEPES, 2 mM L-glutamine, 1 mM sodium pyruvate, and 50 μM β-mercaptoethanol at 37 °C in a humidified atmosphere of 5% CO2, 95% air. Cells were seeded in 96-well plate at a density of 1×10^4 cells per well for 24 h (n=6). Then, the cells were subjected to different interventions. For high-glucose and high-lipid condition, the medium was with 30 mM glucose (HG) and 0.4 mM palmitate (HL). In the HG+HL+Rosiglitazone (R) group, the cells were preincubated in the presence of 10 μM rosiglitazone for 4 h, and subsequently cultured for 24 h in the HG+HL medium. The HG+HL+R+GW9662 group experiment was performed using the same incubation protocol except 10 μM GW9662 addition 2 h earlier. For the HG+HL+GW9662 group, only 10 μM GW9662 was added into the medium without rosiglitazone.

Cell viability after various treatments was determined by CCK-8 (Dojin Laboratories, Kumamoto, Japan). 10 μL of CCK-8 solution (Dojindo Laboratories, Kumamoto, Japan) was

added to each well. After incubation at 37 °C in the incubator for 4 h, the absorbance at 450 nm was monitored by a microplate reader (Synergy Mx, Bio-Tek, USA). Cells incubated with culture medium alone was considered as 100% cell viability.

Consistent with previous reports (Lupi et al., 2004; Han et al., 2008; Wu et al., 2013), exposure to HG+HL reduced viability of INS-1 cell, while pretreatment of rosiglitazone significantly attenuated this effect. Furthermore, treatment of the cell with GW9662 alone has no effect, however, GW9662 markedly diminished the protective effect of rosiglitazone on INS-1 cell (Fig. S2)."

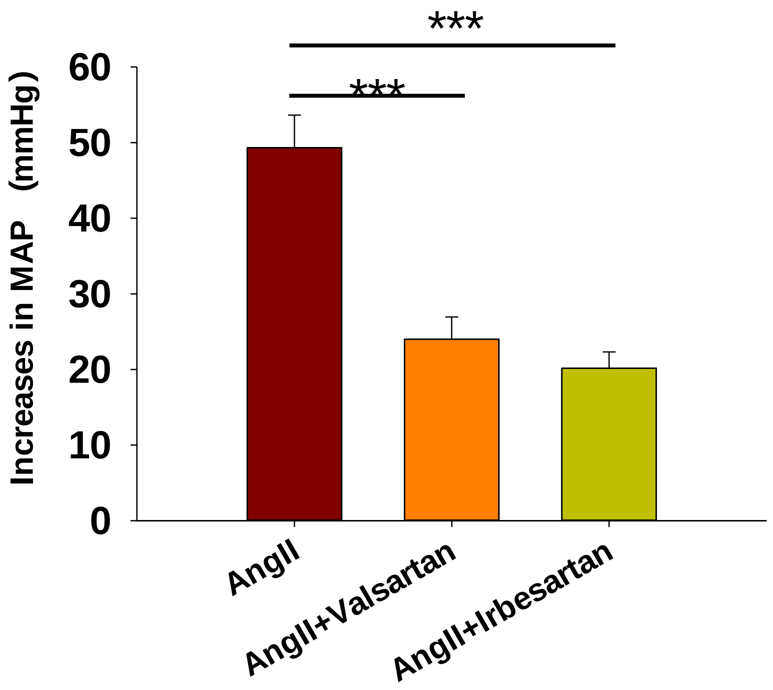

Fig.S1 Pretreatments of the rats with valsartan and irbesartan all reduced the mean arterial pressure (MAP) response to Ang II (200 ng/kg of body weight) injected intravenously (n=6). All results are reported as the means ± SEM. Statistical differences among groups were compared using one-way analysis of variance (ANOVA) and Student–Newman–Keuls method post hoc analysis. *** $p<0.001$.

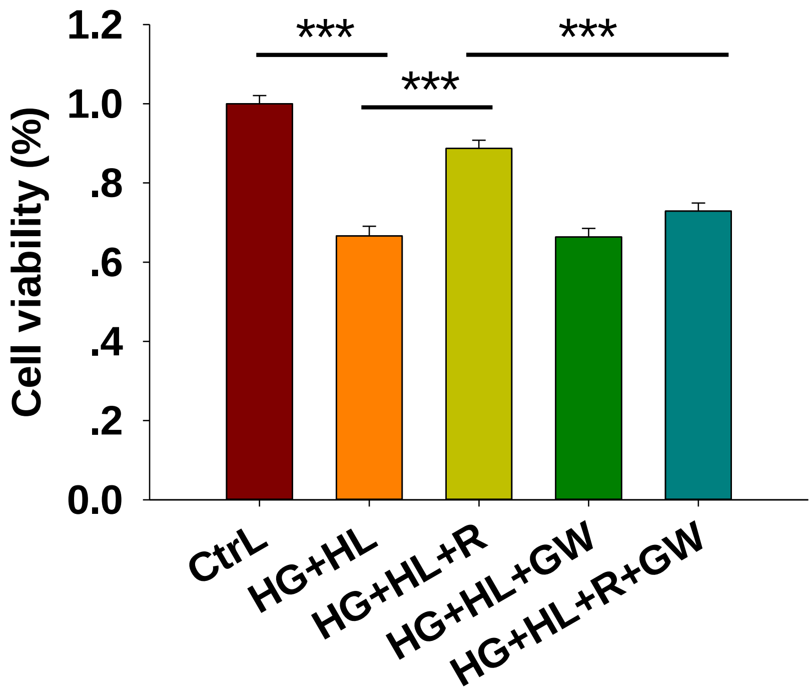

Fig.S2 GW9662 (GW) diminished the protective effect of rosiglitazone (R) on INS-1 cells (n=6). Cell viability was assessed by CCK-8 assay. After different pretreatments, INS-1 cells were exposed to a high-glucose (30 mM) and high-lipid solution (400 μM palmitate) (HG+HL) for 24 h. All results are normalized to control, and reported as the means ± SEM. Statistical differences among groups were compared using one-way analysis of variance (ANOVA) and Student–Newman–Keuls method post hoc analysis. *** $p<0.001$.

REFERENCES

Han, S. J., Kang, E. S., Hur, K. Y., Kim, H. J., Kim, S. H., Yun, C. O., et al. (2008). Rosiglitazone inhibits early stage of glucolipotoxicity-induced beta-cell apoptosis. *Hormone research* 70, 165-173. doi: 10.1159/000137662

Kawai, T., Hirose, H., Seto, Y., Fujita, H., Fujita, H., Ukeda, K., et al. (2002). Troglitazone ameliorates lipotoxicity in the beta cell line INS-1 expressing PPAR gamma. *Diabetes Res. Clin. Pract.* 56, 83-92. doi: 10.1016/s0168-8227(01)00367-9

Lupi, R., Del Guerra, S., Marselli, L., Bugliani, M., Boggi, U., Mosca, F., et al. (2004). Rosiglitazone prevents the impairment of human islet function induced by fatty acids: evidence for a role of PPARgamma2 in the modulation of insulin secretion. *Am. J. Physiol. Endocrinol. Metab.* 286, E560-567. doi: 10.1152/ajpendo.00561.2002

Walter, H., and Lübben, G. (2005). Potential role of oral thiazolidinedione therapy in preserving beta-cell function in type 2 diabetes mellitus. *Drugs* 65, 1-13. doi: 10.2165/00003495-200565010-00001

Wu, J., Wu, J. J., Yang, L. J., Wei, L. X., and Zou, D. J. (2013). Rosiglitazone protects against palmitate-induced pancreatic beta-cell death by activation of autophagy via 5'-AMP-activated protein kinase modulation. *Endocrine* 44, 87-98. doi: 10.1007/s12020-012-9826-5
